# Effekte der gesetzlichen Qualitätssicherung in der akutstationären Versorgung

**DOI:** 10.1007/s00103-022-03489-z

**Published:** 2022-02-04

**Authors:** Max Geraedts, Werner de Cruppé

**Affiliations:** Institut für Versorgungsforschung und Klinische Epidemiologie, Fachbereich Medizin, Philipps-Universität Marburg, Karl-von-Frisch-Str. 4, 35043 Marburg, Deutschland

**Keywords:** Qualität der Gesundheitsversorgung, Qualitätsbewertung, Qualitätsindikatoren, Öffentliche Qualitätsberichterstattung, Überprüfung und Rückmeldung, Quality of healthcare, Quality assessment, Quality indicators, Public reporting, Audit and feedback

## Abstract

**Hintergrund:**

Die gesetzliche Qualitätssicherung (QS) dient der Sicherung und Weiterentwicklung der Qualität der Leistungserbringung. Besonders prominent in Deutschlands akutstationärer Versorgung sind die verpflichtend anzufertigenden Qualitätsberichte (QB) und die Beteiligung an externen Qualitätsvergleichen (eQS). Deren Effekte wurden bisher nicht umfassend evaluiert.

**Fragestellung:**

Mit welchen Effekten auf die Versorgungsqualität gehen eQS und QB einher?

**Material und Methode:**

Auf der Basis einer selektiven Literaturrecherche wurden internationale Erkenntnisse zu den Effekten der QS zusammengestellt. Diese wurden durch Analysen der Qualitätsreporte der seit 2001 für die eQS zuständigen Institutionen, der Bundesgeschäftsstelle Qualitätssicherung (BQS), des Instituts für angewandte Qualitätsförderung und Forschung im Gesundheitswesen (AQUA) und des Instituts für Qualitätssicherung und Transparenz im Gesundheitswesen (IQTIG) ergänzt.

**Ergebnisse:**

Laut internationaler Literatur können höchstens schwache Effekte dieser Maßnahmen vor allem auf die Prozessqualität erwartet werden. Studien aus Deutschland beobachten zumeist nur unkontrolliert zeitliche Trends und weisen zum Teil verbesserte Qualitätsindikatoren nach. Nur je eine kontrollierte Studie konnte für die eQS bzw. die QB jeweils schwache positive Effekte auf die Ergebnis- bzw. Prozessqualität zeigen.

**Diskussion:**

Weder für die QB noch die eQS liegen überzeugende Evaluationsergebnisse vor. Als potenzielle Ursachen können Mängel der adressatenorientierten Gestaltung der QB und der rückgemeldeten Indikatorergebnisse in Bezug auf deren Validität, Risikoadjustierung und zeitliche Verfügbarkeit angeführt werden. Die gesetzliche QS sollte überarbeitet werden, indem die Voraussetzungen für erfolgreiche Leistungsrückkopplungen stärker beachtet werden und wieder Raum für eine intrinsisch motivierte Auseinandersetzung mit der eigenen Versorgungsqualität geschaffen wird.

## Abstract (en)

**Background:**

Statutory quality assurance (QA) serves to ensure and further develop the quality of service provision. Particularly prominent in Germany’s acute inpatient care are mandatory quality reports (QRs) and participation in external quality assessments (eQAs). Their effects have not yet been comprehensively evaluated.

**Objectives:**

What are the effects of eQAs and QRs on the quality of care?

**Materials and methods:**

Based on a selective literature review, international evidence on the effects of QA was compiled. This was supplemented by analyses of the quality reports of the Federal Office for Quality Assurance (BQS), the Institute for Applied Quality Improvement and Research in Health Care (AQUA), and the Institute for Quality Assurance and Transparency in Healthcare (IQTIG), which have been responsible for eQAs since 2001.

**Results:**

According to international literature, at most weak effects of these measures can be expected, especially on process quality. Studies from Germany mostly observe only uncontrolled temporal trends and partly show improved quality indicators. Only one controlled study each was able to show weak positive effects on outcome and process quality for eQAs and QRs, respectively.

**Conclusions:**

There are no convincing evaluation results for either the QRs or the eQAs. Deficiencies in the addressee-oriented design of the QRs and the reported indicator results in terms of their validity, risk adjustment, and temporal availability can be cited as potential causes. Statutory QA should be revised by paying more attention to the prerequisites for successful performance feedback and by again creating room for an intrinsically motivated assessment of one’s own quality of care.

## Einleitung

Das Fünfte Buch Sozialgesetzbuch (SGB V) verpflichtet nach § 135a alle Leistungserbringer „zur Sicherung und Weiterentwicklung der Qualität der von ihnen erbrachten Leistungen“. Darunter fällt für Krankenhäuser die Verpflichtung, einrichtungsintern ein Qualitätsmanagement einzuführen und weiterzuentwickeln, ein patientenorientiertes Beschwerdemanagement einzuführen und sich an einrichtungsübergreifenden Maßnahmen der Qualitätssicherung (QS) zu beteiligen. Diese allgemeinen Forderungen werden vom Gemeinsamen Bundesausschuss (G-BA) in Form von Richtlinien konkretisiert. So beschließt der G‑BA nach § 136b Richtlinien zur QS im Krankenhaus: (1) zu Nachweisen über die Erfüllung der Fortbildungspflichten der Fachärzte und der Psychotherapeuten, (2) zu Mindestmengen für planbare Leistungen, (3) zu Inhalt, Umfang und Datenformat eines jährlich zu veröffentlichenden strukturierten Qualitätsberichts und (4) zu Leistungen oder Leistungsbereichen, zu denen Qualitätsverträge erprobt werden sollen. Bis Juni 2021 galt seit der Änderung des § 136b im Jahr 2016, dass der G‑BA auch (5) Leistungen oder Leistungsbereiche definieren sollte, die sich für eine qualitätsabhängige Vergütung mit Zu- und Abschlägen eignen. Dieser Passus wurde mit dem Gesundheitsversorgungsweiterentwicklungsgesetz (GVWG) jedoch gestrichen.

Unter den genannten gesetzlichen QS-Maßnahmen der akutstationären Versorgung stechen 2 Maßnahmen heraus, die für alle Krankenhäuser mit einem hohen Aufwand einhergehen. Diese Maßnahmen sollen im vorliegenden Beitrag fokussiert werden. Hierbei handelt es sich zum einen um die Qualitätsberichte (QB), die erstmals für das Jahr 2004 und anschließend zunächst zweijährlich anzufertigen waren. Seit 2014 müssen sie jährlich erstellt werden. Zum anderen geht es um die einrichtungsübergreifende, sogenannte externe Qualitätssicherung (eQS). Letztere geht auf die freiwilligen Einrichtungsvergleiche in der Chirurgie und Geburtshilfe zurück, wie sie von Ärzten bzw. Fachgesellschaften seit Anfang der 1970er-Jahre betrieben wurden [1, 2]. Hierbei dokumentieren die Leistungserbringer Daten zu Versorgungsprozessen und -ergebnissen, die zentral zusammengeführt, analysiert und in Form von Leistungserbringervergleichen zurückgemeldet werden. Die Vergleiche können anschließend im Rahmen des internen Qualitätsmanagements genutzt werden, um Verbesserungsmaßnahmen auszulösen.

Seit 1989 verlangte das SGB V eine verpflichtende Teilnahme an der in einzelnen Bundesländern für verschiedene Leistungsbereiche eingeführten eQS. Mit der Einführung der Fallpauschalen und Sonderentgelte für einzelne Prozeduren wurde die eQS für diese Prozeduren ab 1996 bundesweit verpflichtend. Die gesetzlichen Krankenversicherungen befürchteten damals, dass die Einführung dieses Vergütungssystems einen Anreiz für Krankenhäuser darstellt, Gewinne dadurch zu erzielen, dass die Leistungen mit reduzierter Qualität erbracht würden [3]. Ab 2001 wurden viele der zum Teil freiwilligen, auch in nicht von Fallpauschalen erfassten Leistungsbereichen etablierten eQS-Maßnahmen für alle Krankenhäuser verpflichtend, sodass in insgesamt 31 Leistungsbereichen eQS-Daten zu dokumentieren waren. Die administrative Abwicklung der eQS verantworten in den Bundesländern die (Landes‑)Projektgeschäftsstellen bzw. heute Landesarbeitsgemeinschaften und auf der Bundesebene zunächst die Servicestelle Qualitätssicherung (SQS), dann die Bundesgeschäftsstelle Qualitätssicherung (BQS), das Institut für angewandte Qualitätsförderung und Forschung im Gesundheitswesen (AQUA) und seit 2015 das Institut für Qualitätssicherung und Transparenz im Gesundheitswesen (IQTIG).

Im Juni 2021 wurde die Richtlinie über Maßnahmen der QS in Krankenhäusern (QSKH-RL) durch die Richtlinie zur datengestützten einrichtungsübergreifenden QS (DeQS-RL) abgelöst. Diese beschreibt insgesamt 15 bundesweit verpflichtende Verfahren der eQS, die nun zum Teil nicht nur den stationären, sondern auch den ambulanten Sektor betreffen. Die Verfahren reichen von der Dekubitusprophylaxe, die alle Patienten ab 20 Jahren erfasst, über perkutane Koronarinterventionen mit sektorgleicher Gültigkeit bis hin zur Transplantationsmedizin, die nur wenige Krankenhäuser durchführen (Infobox 1).

### Infobox 1: Verfahren der externen Qualitätssicherung laut Richtlinie zur datengestützten einrichtungsübergreifenden Qualitätssicherung (DeQS-RL) 2021

- 1 – Perkutane Koronarintervention (PCI) und Koronarangiographie (QS PCI)
- 2 – Vermeidung nosokomialer Infektionen – postoperative Wundinfektionen (QS WI)
- 3 – Cholezystektomie (QS CHE)
- 4 – Nierenersatztherapie bei chronischem Nierenversagen einschließlich Pankreastransplantationen (QS NET)
- 5 – Transplantationsmedizin (QS TX)
- 6 – Koronarchirurgie und Eingriffe an Herzklappen (QS KCHK)
- 7 – Karotis-Revaskularisation (QS KAROTIS)
- 8 – Ambulant erworbene Pneumonie (QS CAP)
- 9 – Mammachirurgie (QS MC)
- 10 – Gynäkologische Operationen (QS GYN-OP)
- 11 – Dekubitusprophylaxe (QS DEK)
- 12 – Versorgung mit Herzschrittmachern und implantierbaren Defibrillatoren (QS HSMDEF)
- 13 – Perinatalmedizin (QS PM)
- 14 – Hüftgelenkversorgung (QS HGV)
- 15 – Knieendoprothesenversorgung (QS KEP)

Beginnend mit dem Jahr 2001 waren eQS-Dokumentationen zu zunächst 10 %, ab 2002 zu 20 % der damals rund 16 Mio. Krankenhausfälle anzufertigen. Im Jahr 2019 waren im akutstationären Sektor rund 3 Mio. Fälle dokumentationspflichtig, also rund 16 % aller Krankenhausfälle [4], wobei allein die Dokumentation bei den Leistungserbringern im Durchschnitt 15 min Arbeitszeit pro Fall benötigt [5].

Theoretisch fußen sowohl die eQS als auch die Qualitätsberichte implizit auf der sozialkognitiven Lerntheorie [6], also dem Lernen am Modell: Die Rückmeldung der eigenen Leistung im Vergleich zur Leistung anderer bzw. das Bewusstsein über die Transparenz der eigenen Leistungen und die damit verbundenen Vergleichsmöglichkeiten für Patienten, ein- und überweisende Ärzte sowie Kostenträger soll die Leistungserbringer dazu motivieren, am Modell zu lernen, sich also an den jeweils besseren Leistungserbringern zu orientieren. Davon abgeleitet postulierten Berwick et al. [7] 2 Wirkmechanismen von Vergleichsinformationen: Adressaten wählen auf der Basis der Vergleichsinformationen die jeweils besten Leistungserbringer aus („selection pathway“) oder die Leistungserbringer ändern die Versorgung, da sie zu Veränderungen bzw. zur Qualitätsverbesserung motiviert werden, wenn sie sich selber als schlechter im Vergleich zu anderen erkennen („change pathway“). Im Unterschied zur international üblichen Rückmeldung von Leistungsergebnissen auf der Basis unabhängig erhobener Daten („audit and feedback“) beruht die Rückmeldung in Deutschland jedoch auf selbstberichteten Daten, deren Validität im Rahmen der eQS nur zum Teil überprüft wird.

Dennoch geht der Gesetzgeber laut Begründung zum GVWG davon aus, dass die Transparenz auf Grundlage der eQS und Qualitätsberichte zu Auswahl und Veränderung führe und zu erwarten sei, „… dass sich die Struktur‑, Prozess- und Ergebnisqualität bei den Leistungserbringenden mit der Neuregelung weiter stetig verbessern werden“ [8].

Ob sich diese hohen Erwartungen der Bundesregierung an die für alle Leistungserbringer aufwendigen Maßnahmen in der Versorgungsrealität erfüllen werden, ist fraglich. Um sich einer Beantwortung der Frage anzunähern, werden im Folgenden die bislang vorliegenden Erkenntnisse zu den Effekten dieser beiden gesetzlichen Qualitätssicherungsmaßnahmen in der akutstationären Versorgung auf der Basis einer selektiven Literaturanalyse und aktueller Forschungen der Autoren zusammengestellt.

## Material und Methode

PubMed wurde anhand der Suchbegriffe „german*“, „quality assurance“, „audit and feedback“, „public reporting“, „quality report*“ durchsucht; davon ausgehend wurde die jeweils zitierte Literatur analysiert sowie graue Literatur anhand von Internetrecherchen, vor allem bei den jeweils für die QS zuständigen Institutionen ergänzt. Im Besonderen wurden die Qualitätsreporte zur eQS seit 2001 von BQS, AQUA und IQTIG herangezogen.

## Ergebnisse

### Erkenntnisse aus der internationalen Literatur

Die internationale Literatur zu den Grundprinzipien der in der Gesundheitsversorgung eingesetzten Maßnahmen der Qualitätsförderung wurde kürzlich in der OECD-Publikation *Improving healthcare quality in Europe – Characteristics, effectiveness and implementation of different strategies* zusammengefasst [9]. Zudem finden sich zu den hier angesprochenen QS-Maßnahmen der akutstationären Versorgung in Deutschland 2 Cochrane-Reviews. Zur Qualitätsberichterstattung (QBE) folgern die Autoren in dem zuletzt 2018 aktualisierten Cochrane-Review [10], dass die existierende Evidenz unzureichend ist, um diese Maßnahme politisch zu befürworten: In einzelnen Studien wird auf der Basis unsicherer Evidenz kein oder höchstens ein geringer Einfluss zum einen auf Auswahlentscheidungen von Patienten, ein- oder überweisenden Ärzten und Kostenträgern und zum anderen auf Gesundheitsergebnisse festgestellt. Die Autoren des OECD-Buchs berichten ebenfalls über eine geringfügige Sterblichkeitsreduktion in einzelnen Studien zur Wirkung der QBE, wobei diese Ergebnisse eher in unkontrollierten Studien und in Bezug auf die kardiovaskuläre Sterblichkeit zu sehen waren und in Fällen, bei denen die Ausgangsqualität niedrig war [11]. Zudem weisen die Autoren auf Nebeneffekte der QBE wie eine veränderte Codierung oder Wiedereinweisungspraxis hin.

Etwas positiver fällt das Urteil für das Prinzip des „audit and feedback“ aus. Hier kommt das zuletzt 2012 aktualisierte Cochrane-Review zum Fazit, dass dieses Prinzip mit geringen, aber möglicherweise wichtigen Verbesserungen der klinischen Praxis einhergeht [12]. Die Rückmeldungen werden als besonders wirksam eingeschätzt, wenn die Qualität der Versorgung zu Beginn niedrig ist und die Rückmeldungen durch Kollegen, mehrfach und auf verschiedene Weise erfolgen sowie mit Zielen und einem Aktionsplan verbunden sind. Die gleiche Autorengruppe fasst die Erkenntnisse im OECD-Buch 2019 so zusammen, dass „audit and feedback“ moderate Effekte auf die Konformität mit einer gewünschten klinischen Praxis zeigt, die Evidenz für Effekte auf die Versorgungsergebnisse aber nicht klar sei, obwohl einige Studien positive Effekte berichten [13]. Zudem weisen die Autoren darauf hin, dass oftmals verschiedene Maßnahmen der Qualitätsförderung – wie „audit and feedback“, öffentliche Qualitätsberichterstattung, Akkreditierung bzw. Zertifizierung und qualitätsabhängige Vergütung – kombiniert eingesetzt werden und die Effekte der einzelnen Elemente schwer zu trennen sind.

### Erkenntnisse zur Ergebnisrückmeldung aus Deutschland

Die internationale Literatur lässt sich so zusammenfassen, dass im positiven Fall höchstens geringe Effekte zu erwarten sind. Wie steht es aber nun um Erkenntnisse aus Deutschland?

Hierzu liegen eine Überblicksarbeit und eine Reihe einzelner Studien vor. Im Review von Khan und Ollenschläger [14] finden sich Hinweise auf positive Effekte einzelner QS-Maßnahmen in Deutschland, jedoch nicht für die gesetzliche eQS, deren besonderes Kennzeichen darin besteht, dass sie jeweils ohne Vorstudien bundesweit eingeführt wurde. Studien zur eQS verfolgen typischerweise die Ausprägung von Indikatoren über die Zeit und weisen für einzelne Leistungsbereiche oder über Leistungsbereiche hinweg zum Teil positive Entwicklungen nach [15–17]. Andere Studien aus Deutschland konnten zeigen, dass positive Ausprägungen von Prozessqualitätsindikatoren mit positiven Ergebnissen für Patienten assoziiert sind. Beispielhaft genannt werden sollen hierzu aktuelle Analysen aus dem Traumaregister, einem klinischen Krebsregister und den Schlaganfallregistern, die jedoch alle nicht zu den verpflichtenden QS-Maßnahmen zählen [18–20]. Aber auch anhand von Daten der eQS konnte gezeigt werden, dass positive Indikatorausprägungen mit besseren Versorgungsstrukturen und auch besseren Patientenerfahrungen einhergehen [21]. Diese Studien belegen jedoch letztlich nur, dass die Struktur- oder Prozessqualitätsindikatoren richtig ausgewählt wurden, da tatsächlich ein positiver Zusammenhang zu Versorgungsergebnissen existiert.

**Figure 1:**
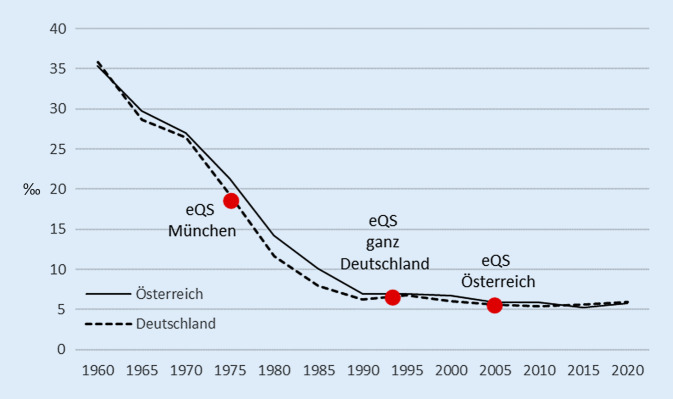

Problematisch bei den Studien ist im Allgemeinen, dass sie nicht kontrolliert sind und somit eine Kausalität der Beziehung aufgrund der Studiendesigns kaum abzuleiten ist. Gerade im Gesundheitsbereich kann dies besonders nachteilig wirken, weil hier oftmals weitere Interventionen Einfluss nehmen, sodass zeitliche Trends schwierig zu interpretieren sind. Abb. 1 verdeutlicht das Problem an der perinatalen Sterblichkeit im Vergleich für Deutschland und Österreich. Misst man die Qualität der Geburtshilfe an diesem Indikator, dann ergibt sich für beide Länder eine fulminante Verbesserung von rund 35 pro 1000 Lebend- und Totgeborenen in 1960 auf knapp 6 pro 1000 im Jahr 2020. Dies allein auf die eQS „Perinatalerhebung“ zurückzuführen, die zunächst ab 1975 in München, dann in immer mehr Bundesländern und ab 1993 in ganz Deutschland sowie ab 2005 als Geburtenregister in Österreich eingeführt wurde, wäre in Anbetracht des zeitlichen Trends sicher nicht richtig. Stattdessen hat sich in der beobachteten Zeitspanne die „Umwelt“ der Versorgung in vielerlei Hinsicht verändert: Unter anderem die Geburtshilfe, die Lebensverhältnisse und die Nennerdefinition (seit 1994 zählen Totgeborene ab 500 g Geburtsgewicht, vormals erst ab 1000 g) haben sich verändert und einen Beitrag zum Trend geleistet. Wie hoch der Beitrag der eQS war, lässt sich bei alleiniger Betrachtung des Trends nicht quantifizieren.

Vor diesem Hintergrund hat das kürzlich abgeschlossene Innovationsfondsprojekt „QUASCH – Ergebnisse qualitätsgesicherter Schlaganfallversorgung – Hessen im Vergleich zum übrigen Bundesgebiet“ (FKZ 01VSF18041) die Tatsache ausgenutzt, dass bei der Schlaganfallversorgung nur einzelne Bundesländer eine verpflichtende eQS eingeführt haben, während andere Bundesländer ohne eQS blieben und als Kontrollgruppe dienen konnten. Im Projekt wurde unter anderem das Sterblichkeitsrisiko von Schlaganfallpatienten im Zeitraum 2007–2017 analysiert. Dabei konnte erstmals eine risikoadjustierte Sterblichkeitsreduktion in Bundesländern mit eQS im Vergleich zu Bundesländern ohne eQS festgestellt werden, wobei alle Bundesländer im Zeitverlauf ansonsten vergleichbare Rahmenbedingungen erfahren hatten [22]. Deutlich wurde in der Studie aber auch, dass der eQS-Einfluss auf das Sterblichkeitsrisiko im Verlauf der Jahre sank und dass die Versorgung in einer Spezialeinheit für Schlaganfallpatienten, der sog. Stroke Unit, mit einer wesentlich stärkeren Risikoverminderung einherging.

### Analysen der Qualitätsreporte

Der QUASCH-Studie gelang also die Berücksichtigung der Kontextveränderungen, die ansonsten die Evaluation von Effekten der eQS erschweren. Aber nicht nur Änderungen des Kontexts der eQS sind bei deren Evaluation zu berücksichtigen. Auch die eQS selber hat sich stetig weiterentwickelt. Die Zahl der einbezogenen Leistungsbereiche betrug in der Spitze 30, durch Aussetzen einzelner und Zusammenfassung anderer Bereiche sind die oben aufgeführten, aktuell gültigen 15 Verfahren verblieben. Innerhalb der Verfahren werden Qualitätsindikatoren (QI) betrachtet, die ebenfalls jährlich Anpassungen unterlagen. In der Spitze wurden im Jahr 2012 Daten zu 464 QI erhoben, 2019 waren es 289 QI. Diese Reduktion kam größtenteils durch eine vom IQTIG so bezeichnete „Systempflege“ zustande, bei der QI, für die keine Referenzbereiche definiert waren, nur noch als Kennzahlen bezeichnet werden. Mit der Reduktion der Anzahl beobachteter QI ging auch der Anteil der QI zurück, bei denen im Jahresvergleich keine Veränderung der Indikatorausprägung festzustellen war.

**Figure 2:**
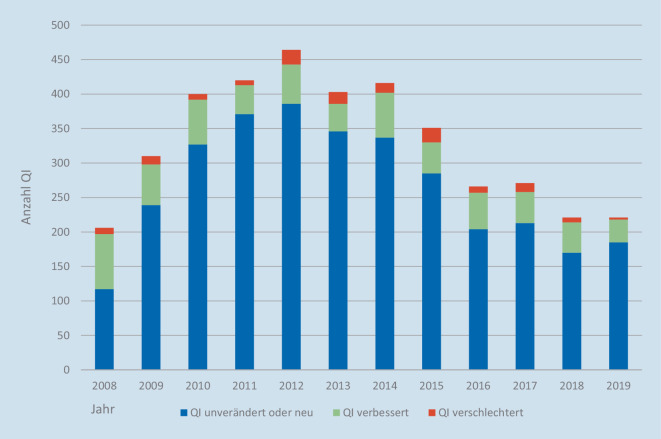

Abb. 2 verdeutlicht für den Zeitraum 2008–2019 die in den Qualitätsreports von BQS, AQUA bzw. IQTIG als im jeweiligen Vorjahresvergleich unverändert/neu, verbessert oder verschlechtert angegebene Anzahl an QI. Mit Ausnahme des Jahres 2008 lag die Anzahl verbesserter QI im Durchschnitt bei 40–60, die Anzahl verschlechterter QI bei 7–20. Im letzten Beobachtungsjahr 2019 waren nur 33 QI besser und 3 QI schlechter im Vergleich zum Vorjahr, der Rest der QI blieb unverändert oder war neu eingeführt worden. Durchschnittlich verbleiben rund 80 % der QI in der eQS zumindest auf dem Vorjahresniveau, zum Teil verharren die QI seit Jahren auf dem gleichen Niveau.

Zusätzlich zur Zahl der QI veränderte sich mit den Jahren auch die Risikoadjustierung bei den pro Krankenhaus betrachteten Populationen, indem bei mehr QI multivariate Modelle zur Risikoadjustierung eingesetzt werden. Inzwischen gibt das IQTIG an, dass rund 25 % der QI risikoadjustiert sind. Dass die Risikoadjustierung in der gesetzlichen eQS insgesamt nicht nur quantitativ, sondern auch qualitativ noch unzureichend zu sein scheint, macht eine kürzlich vorgelegte Analyse des Zusammenhangs zwischen Krankenhausstrukturvariablen und den QI-Ausprägungen deutlich. Demnach erzielen in Deutschland über eine Vielzahl der QI hinweg gerade diejenigen Krankenhäuser mit den niedrigsten Fallzahlen die besten Qualitätsergebnisse. Dieses kontraintuitive Ergebnis führen die Autoren auf eine unzureichende Risikoadjustierung zurück [23].

Ein weiterer seit Jahren diskutierter Kritikpunkt betrifft die Validität der selbstberichteten Daten. Die zuständigen Institutionen prüfen dabei die Vollzähligkeit, Vollständigkeit, Plausibilität und Richtigkeit der Daten zum einen durch eine statistische Basisprüfung, der sich bei Auffälligkeit der sogenannte strukturierte Dialog anschließen kann. Zum anderen wird eine Stichprobe von Krankenakten geprüft, die mit den elektronisch übermittelten Daten abgeglichen werden. Beim strukturierten Dialog werden einer Klinik bei rechnerisch auffälligen Ergebnissen entweder nur Hinweise gegeben oder es wird um eine Stellungnahme gebeten. Wenn diese den Sachverhalt nicht klären kann, können Zielvereinbarungen getroffen oder sogar Begehungen durchgeführt werden, die letztlich zum Entzug der Abrechnungsfähigkeit von Leistungen führen können.

Zu den Validierungen mithilfe von Stichprobenprüfungen sowie dem strukturierten Dialog liegen Erkenntnisse der verantwortlichen Institutionen und aus einzelnen Studien vor. So ergaben die Stichprobenprüfungen mit Datenabgleich der eQS-Dokumentationen mit den Patientenakten, die jeweils 3 wechselnde Leistungsbereiche, 5 % der Krankenhausstandorte und hier bis zu 20 Krankenhausfälle umfassen, in den letzten Jahren zusammenfassend laut den Qualitätsreporten von BQS, AQUA und IQTIG folgendes Ergebnis: Während die BQS in den Jahren 2006 und 2007 über Raten zwischen 0 % und 100 % Übereinstimmung zwischen den Angaben zu einzelnen Datenfeldern in den Krankenakten und den elektronisch übermittelten Daten berichtete, gab es vom AQUA-Institut nur eine Kategorisierung in „verbesserungsbedürftig“, „gut“ oder „hervorragend“, wobei aus den Jahren 2009 und 2010 zwischen 27–64 % der geprüften Datenfelder als verbesserungsbedürftig eingestuft wurden. Daten zu Prozeduren stimmten laut AQUA eher überein als solche zu Komplikationen. Das IQTIG gibt für die Jahre 2016–2019 Übereinstimmungsraten zwischen 58 % und 100 % an, wobei die Raten im Durchschnitt über alle geprüften Leistungsbereiche und Krankenhäuser hinweg über 90 % lagen.

Vergleichbare Ergebnisse erbrachten auch Studien in einem einzelnen Krankenhaus, bei dem die Krankenakten mit den eQS-Daten verglichen wurden [24, 25], sowie der Vergleich von Daten der fallpauschalenbezogenen Krankenhausstatistik (DRG) mit den eQS-Daten [26]. Beide Evaluationsansätze zeigten, dass sich die Datensätze signifikant unterschieden und insbesondere Komplikationen in den eQS-Daten unterdokumentiert vorkamen.

Der strukturierte Dialog, der ebenfalls lange in der Kritik stand, weil er in den Bundesländern unterschiedlich gehandhabt wurde, wurde im Rahmen der DeQS-RL aktuell unter der Bezeichnung „Stellungnahmeverfahren“ vereinheitlicht. Daher liegen noch keine neuen Evaluationsergebnisse vor. Bisher erbrachte das für alle Beteiligten aufwendige Verfahren regelmäßig, dass nur 10–15 % der rechnerisch auffälligen Ergebnisse, die in den weiteren Prozess eingeschleust wurden, letztlich als qualitativ auffällig eingestuft wurden. Die Tab. 1 gibt beispielhaft die Rate der auffälligen Ergebnisse aus den aktuellsten, gleichförmig berichteten Ergebnissen der Erfassungsjahre 2016–2018 wieder. Regelmäßig wird auch berichtet, dass relevante Dokumentationsmängel eine Beurteilung unmöglich machen.

**Table 1:** 

–	Anzahl der Datensätze	Einbezogene Qualitätsindikatoren (QI)	Berechnete Ergebnisse	Rechnerisch auffällig	Qualitativ auffällig	Dokumentationsmängel
2016	2.482.141	219	116.163	12.683 (10,9 %)	1611 (12,7 %)	961 (7,6 %)
2017	2.495.813	214	110.662	11.413 (10,3 %)	1465 (12,8 %)	852 (7,5 %)
2018	2.479.366	202	98.782	9998 (10,1 %)	1482 (14,8 %)	568 (5,7 %)

Diese Ergebnisse lassen Zweifel an der Effizienz des Verfahrens aufkommen. In Anbetracht weiterer Analysen [27], die belegen, dass der Großteil der qualitativen Auffälligkeiten auf wenige Qualitätsindikatoren zurückzuführen ist, muss zudem die Datengrundlage selber, also die Güte der einbezogenen Indikatoren, hinterfragt werden. Gleichzeitig bieten diese Ergebnisse Ansatzpunkte für eine Verschlankung dieses Verfahrens durch eine Konzentration auf problematische QI.

### Erkenntnisse zu den gesetzlichen Qualitätsberichten

Ebenso wie die QI der eQS haben sich die gesetzlichen Qualitätsberichte selber als Ausgangspunkt für Transparenz und darauf aufbauende Auswahlentscheidungen seit ihrer Einführung im Jahr 2004 in Bezug auf die Datengrundlage sowie deren öffentliche Zugänglichkeit verändert. Dabei ist besonders hervorzuheben, dass der Öffentlichkeit bis zum Jahr 2005 Daten zur Qualität der Versorgung in Form von Qualitätsindikatorausprägungen zu Prozessen und Ergebnissen der Versorgung nur in aggregierter Form zur Verfügung gestellt wurden, seit 2006 jedoch die Daten zur Ausprägung von Qualitätsindikatoren pro Krankenhaus in den gesetzlichen Qualitätsberichten veröffentlicht werden. Die Zahl der veröffentlichungspflichtigen Indikatoren stieg dabei von 30 auf 295 im Jahr 2013 und geht seitdem parallel zur Reduktion der in der eQS erhobenen Qualitätsindikatoren wieder zurück.

Studien zur Wirkung der Qualitätsberichte in Deutschland sind zu allen 3 Adressatengruppen (Patienten, ein- und überweisende Ärzte, Krankenhäuser) durchgeführt worden und ergeben ein insgesamt ernüchterndes Bild. Demnach kennen die meisten Patienten die Berichte kaum, nutzen diese nicht und zeigen, wenn man sie mit den Berichten konfrontiert, Verständnisprobleme [28–30]. Ähnlich sieht es bei niedergelassenen, ein- und überweisenden Ärzten aus; auch diese kennen und nutzen die Qualitätsberichte der Krankenhäuser nur in geringem Umfang [31]. Die Krankenhäuser selber scheinen die Berichte hingegen zu beachten. So konnten Kraska et al. [32] mithilfe eines kontrollierten Prä-Post-Designs feststellen, dass sich nach der Einführung der verpflichtenden öffentlichen Berichterstattung genau diejenigen Qualitätsindikatoren verbesserten, die krankenhausbezogen publiziert wurden, während andere, nicht öffentlich berichtete Indikatoren keine solche Verbesserung aufwiesen.

## Diskussion

Die in Deutschland in der akutstationären Versorgung eingeführten gesetzlichen Qualitätssicherungsmaßnahmen der sogenannten externen Qualitätssicherung sowie Qualitätsberichterstattung wurden – trotz ihrer langjährigen Einführung sowie des großen damit verbundenen Aufwands für die Leistungserbringer und die Selbstverwaltung – bisher nur unzureichend evaluiert. Vorliegende Erkenntnisse der meisten Studien aus Deutschland sowie der für die administrative Abwicklung der Maßnahmen zuständigen Institutionen sind aufgrund ihres unkontrollierten Designs nicht in der Lage, potenzielle Effekte von zeitlichen Trends zu differenzieren [1, 2, 4, 5, 14–17]. Die wenigen Studien mit einem kontrollierten Design bestätigen die Ergebnisse der internationalen Literatur zu den möglichen Effekten öffentlicher Berichterstattung und Rückmeldung von Leistungsdaten [9–13]. Demnach erzielen die eingeführten qualitätssichernden Maßnahmen in Deutschland wie zu erwarten nur schwache Effekte im Hinblick auf eine Verbesserung von Gesundheitsergebnissen der Patienten [22, 28–33].

Welche potenziellen Gründe lassen sich für die schwachen Effekte der qualitätssichernden Maßnahmen anführen? Für die Qualitätsberichterstattung ist zunächst die zwar leicht wachsende, jedoch immer noch geringe Bekanntheit der Berichte zu nennen. Zudem empfinden die meisten Adressaten die Berichtsinhalte als nicht zielgerichtet informativ. Darüber hinaus dominiert weiterhin keine auf objektiven Informationen beruhende, sondern eine beziehungsbetonte Krankenhausauswahlentscheidung [28–31, 34].

In Bezug auf die Effekte der Ergebnisrückkopplung im Rahmen der externen Qualitätssicherung muss vor allem die Datengrundlage beachtet werden. Die selbstberichteten Daten haben weiterhin eine eingeschränkte Validität [25, 26], die berechneten Indikatoren sind größtenteils unzureichend risikoadjustiert [23], die Indikatoren weisen zumeist nur noch ein geringes Verbesserungspotenzial auf und nur wenige Indikatoren werden letztlich als qualitativ auffällig beurteilt [27]. Hinzu kommt, dass die Rückmeldung erst mit einer Verzögerung von meist 1–2 Jahren nach der jeweiligen Patientenbehandlung erfolgt, wodurch die Handlungsrelevanz vermindert wird. Dazu hat der G‑BA zwar kürzlich eine vierteljährliche Rückmeldung beschlossen, diese bleibt aber aktuell aufgrund der Coronapandemie ausgesetzt. Allgemein haben die theoretische Untermauerung der Qualitätsrückmeldung und darauf aufbauend die Berücksichtigung derjenigen Faktoren, die die Wirkung von Rückmeldungen zu medizinischen Leistungsdaten beeinflussen, in Deutschland bisher wenig Beachtung gefunden. Zu nennen sind vor allem die Fähigkeit der Adressaten, die Daten zu interpretieren und in Verbesserungen umzusetzen, deren Meinung zur Glaubwürdigkeit der Daten, deren Motivation, die Art der Rückkopplung und Kontextfaktoren wie die Qualitätskultur des jeweiligen Krankenhauses [35, 36].

Als Limitation der vorliegenden Literaturanalyse ist zum einen die nicht systematische, sondern selektive Methodik zu nennen, die aufgrund des bekanntermaßen begrenzten Literaturfundus gewählt wurde. Zum anderen weisen die Hauptquellen der Analysen, die Qualitätsreporte von BQS, AQUA und IQTIG, durchgängig Inkonsistenzen in den Berichten auf, nicht nur zwischen diesen verschiedenen, im Verlauf der Jahre zuständigen Institutionen, sondern es bestehen ebenso innerhalb der Institutionen von Jahr zu Jahr Unterschiede in der Ergebnisaufbereitung, wodurch eine longitudinale Analyse der Rückmeldeeffekte erschwert wird.

## Fazit

Um die Effektivität der qualitätssichernden Maßnahmen in Deutschland in Zukunft zu steigern, könnten folgende Empfehlungen hilfreich sein:

- Beachtung der theoretischen Fundierung der Leistungsrückkopplung,
- mehr zeitnahe Hilfestellung und Kooperation mit den Adressaten, anstatt einer verzögerten Datenrückmeldung in Verbindung mit einem ineffizienten Stellungnahmeverfahren,
- Beachtung der Binsenweisheit, dass Maßnahmen der Qualitätssicherung nicht ohne eine Erprobung in Studien und eine begleitende Implementationsforschung in der Routine eingeführt werden sollten,
- Berücksichtigung ergänzender Möglichkeiten zur Qualitätsförderung, wie die stärkere Einbeziehung von Strukturvorgaben, regionale Absprachen zur Versorgung und dabei insbesondere die Beachtung der Personalausstattung und -motivation, welche im Zuge der Coronapandemie bereits eine höhere Aufmerksamkeit erlangt haben.

Langfristig ist zu hoffen, dass sich dadurch – wie zu Beginn der externen Qualitätssicherung in Deutschland – wieder Raum für die intrinsisch motivierte Auseinandersetzung mit der eigenen Behandlungsqualität ergibt, die auf der Basis einer etablierten Qualitätskultur eine sichere und gute Patientenbehandlung ermöglicht.
